# Comment on "Determination of risk factors in non-obstructive coronary artery myocardial ınfarction"

**DOI:** 10.1590/1806-9282.20251509

**Published:** 2026-05-01

**Authors:** Hüseyin Durmaz

**Affiliations:** 1 Konya City Hospital – Konya, Turkey.

Dear Editor,

I read with interest the article "Determination of Risk Factors in Non-Obstructive Coronary Artery Myocardial Infarction"^1^ by Dr. Dursun et al., recently published in the journal Revista da Associação Médica Brasileira. This study is a significant study in determining the risk factors and possible causes of non-obstructive coronary artery myocardial infarction. The data presented in the study clearly demonstrate the possible pathophysiology of non-obstructive coronary artery myocardial infarction (MINOCA). In this sense, understanding the causes of MINOCA and the recommendations offered for its prevention is quite important. However, I believe there are some points that need to be addressed in this study. The importance of the proximal or distal location of the muscular bridge in the vessel for MINOCA is undeniable. The study does not provide information regarding the location of the muscular bridge. There are two types of C-reactive protein (CRP): standard CRP and high-sensitivity CRP (hs-CRP). hs-CRP is considered a marker of mild, low-grade vascular inflammation. This study did not provide information on the type of CRP used. Vitamin D synthesis, absorption, and metabolism in the body are affected by many factors, including sun exposure, geographic location, amount of skin pigmentation, age, and diseases (obesity, cystic fibrosis, and celiac disease). This study did not achieve homogeneity in the vitamin D-deficient group, except for geographic location. In conclusion, Dr. Dursun et al.'s study addresses an important issue related to MINOCA and offers a valuable perspective. I eagerly await further research in this area and hope the authors will consider these suggestions in their future studies. Thank you for the opportunity to provide feedback on this important study.

## Data Availability

The datasets generated and/or analyzed during the current study are available from the corresponding author upon reasonable request.
